# Complex Economies Have a Lateral Escape from the Poverty Trap

**DOI:** 10.1371/journal.pone.0168540

**Published:** 2017-01-10

**Authors:** Emanuele Pugliese, Guido L. Chiarotti, Andrea Zaccaria, Luciano Pietronero

**Affiliations:** 1 Institute for Complex Systems - CNR, Via dei Taurini 19, 00185, Rome, Italy; 2 Dipartimento di Fisica, Sapienza Università di Roma, P.le Aldo Moro 2, 00185, Rome, Italy; East China University of Science and Technology, CHINA

## Abstract

We analyze the decisive role played by the complexity of economic systems at the onset of the industrialization process of countries over the past 50 years. Our analysis of the input growth dynamics, considering a further dimension through a recently introduced measure of economic complexity, reveals that more differentiated and more complex economies face a lower barrier (in terms of GDP per capita) when starting the transition towards industrialization. As a consequence, we can extend the classical concept of a one-dimensional poverty trap, by introducing a two-dimensional poverty trap: a country will start the industrialization process if it is rich enough (as in neo-classical economic theories), complex enough (using this new dimension and laterally escaping from the poverty trap), or a linear combination of the two. This naturally leads to the proposal of a Complex Index of Relative Development (CIRD) which shows, when analyzed as a function of the growth due to input, a shape of an upside down parabola similar to that expected from the standard economic theories when considering only the GDP per capita dimension.

## Introduction

The study of economic growth targets both the growth of developed, industrialized countries, and the growth of developing countries. While studying the growth of developed countries, most of the growth is seen as an equilibrium process, a trend of continuous growth on a well established path. Both the models, those in which growth is exogenous [1] and those in which it is endogenous [2], can be represented as processes going along an equilibrium path, where the inputs grow along the path following an increases in efficiency (exogenous or endogenous). This focus on equilibrium states really shaped the discipline and gave great attention to the growth of the efficiency in the use of factors, often measured as Total Factor Productivity. Empirical research shows that, indeed, most of the differences in growth among countries are explained by differences in the growth of Total Factor Productivity [3].

However, when considering developing countries this equilibrium view is less justified: using the words of Nelson [4], while *growth* refers to the phenomenon according to which “things simply get bigger”, the idea of *development* refers to a moment where “a lot of qualitative changes are also happening”. One is in fact looking at a tumultuous change, a switch between two different equilibria driven by a sudden spike in investments, both in human and physical capital. The specific source of this driving force pushing the developing countries to a new equilibrium varies with theories, spanning between supply sides explanations [1] and demand side accounts [5, 6]. Most of those theories are characterized by the presence of a barrier. In order to overcome this barrier a certain endowment of factors of production is required. This then kindle new investments in a positively reinforced avalanche-like dynamics, untapping the hidden potential of the country while new economic activities start. This process is known as the *escape from the poverty trap*, and it will be the focus of this paper.

Regarding the industrialization process these neo-classical theories are clearly at odds with empirical data. For instance, the growth of India started in the first half of the ’90s, when the country was at an historical minimum in its income and wealth endowment. Moreover any of the Asian Tigers was much poorer than any South American country when they started their striking development in the late ’60s. In this paper, we try to reconcile theory and empirical data by exploring the nature of the barrier to industrialization together with the presence of unobservable endowments of capabilities, intangible inputs, possibly related to the complexity of the economy at the onset of the industrialization process.

Indeed, along the path toward industrialization, the industrial capabilities of the country, the corresponding products, and consumer preferences are completely reorganized, leading to the population’s freedom to pursue their own interests in unexpected (new) sectors and entrepreneurial activities. This fact dramatically increases the diversification and the complexity of the underlying economy. We quantitatively measure the complexity of an economy through a new dimension, the fitness of the country, that has recently been introduced in the study of social and economic systems [7]. Economic systems share with traditional complex systems [8] the emergence of unexpected collective behaviors coming from non-trivial interactions between their basic components. In our view the industrialization of a country is a dynamic process in which a complex network reinforcing production capabilities and product demand emerges at the country scale. The prosperity and the potential of a country can then be characterized by considering this new dimension [9], which takes into account the diversification and the complexity of the production system.

In our analysis we look at the empirical growth patterns of countries having different levels of fitness. As will emerge, high country fitness is associated with a lower barrier toward the industrialization of that country, acting as a unobservable endowment critically affecting the threshold to be overcome in order to start the industrialization process. Moreover we show that, once we correct the poverty trap models to consider unobservable endowments, the theory is able to predict the industrialization of countries.

The following paper will be divided in four sections in addition to this introduction. In the Materials and Methods section in order to fix the notations, we will briefly explain the classic poverty trap and growth accounting, and we will then devote a subsection to describe the economic complexity approach. In the Results section we will show some empirical shortcomings of this basic idea of poverty trap and we will show the promising role of our Fitness index to solve these shortcomings. We will then show that introducing Fitness in the basic poverty trap narrative helps the theory to describe the empirical evidence. In the Discussion section we will sum up our results, suggest future research and exploit these empirical findings to obtain suggestions for the policy makers. The data used for our analysis and a more in depth discussion of the theoretical poverty trap approach is described in the Supporting Information.

## Materials and Methods

### The Neoclassical Economic approach to Growth

#### The poverty trap in standard economic theory

In most of the countries that will eventually join the other developed nations, the early stages of economic growth are characterized by a period of fast growth. Well known examples comprise the growth of the Soviet Union [10], the growth of Japan and Southern Europe in the ’50s, the growth of the Asian Tigers in the ’70s: every country emerging from an agriculture based economy has experienced a decade or more of extremely high growth while it is catching up with the other developed countries. The spike of high growth is characterized by a strong increase in investments, both in physical and human capital. While the population experiences new incentives and opportunities for education and investment, the factors of production rise inflating economic growth. This is what we define as industrialization: the moment in which there is a sudden spike in the factors of production available in the country.

Following the influential work of Solow [1], this sudden transition from mere subsistence to complete industrialization has been described with the presence of a trap that could be avoided by overcoming a barrier in terms of wealth, or physical capital. More formally, the usual picture makes use of the presence of multiple equilibrium paths, characterized by different equilibrium ratios of capital and labor, in the time evolution of physical capital. In this picture, the presence of multiple equilibrium paths is due to the non linear relation between the country investments in physical capital and GDP due to the presence of a threshold in the saving rate. This dynamic evolution results in a non linear increase of the available capital at successive times. The main ideas behind the simple Poverty Trap model [1] are presented in the Supporting Information.

This kind of explanation, being related to investments, is said “supply side”, since it implies that the lack of enough investments is the missing variable for achieving industrialization. In this picture what is missing to inject growth are plants able to produce more profits to be reinvested again: overcoming a threshold level of physical capital is necessary to start the expansive cycle. There are many competing explanations however, typically marked as “demand side”, where the lacking variable is enough internal demand to kick-start industrialization, as in [5, 6]. In this kind of explanation, often referred as “The Big Push”, one sector industrializing increases the wages of part of the population, that requires more goods and, therefore, pushes for the industrialization of other sectors in a cascade dynamics. While to address the issue of these different competing narratives is an important research question, it is not the question we want to answer in the current analysis.

Any explanation, both on the demand and the supply side, can therefore be assumed as long as it allows for a threshold of resources needed to trigger industrialization. Since GDP per capita or average wage—required as a threshold by a demand side model of poverty trap with increasing returns—and the physical capital—required as a threshold by a traditional model of poverty trap—correlate very strictly (in the Penn World Table 8.0 [11], described in the Supporting Information, the Spearman’s rank correlation between GDP and physical capital is 96.2% over the period 1963–2000), we will be able to keep the same fuzziness also in the next, more empirical, analysis. Economic theory is used in our empirical analysis only to separate the contribution to growth due to innovation from that due to inputs, as illustrated in the next section.

#### Discerning the share of GDP growth due to change in inputs

To understand the role played by the complexity of the economy in the industrialization process we brake down the relative growth of output of the country’s production structure (GDP) into its main components: the one related to variations of technological efficiency and those related to variations of inputs, namely asset capital and human capital. This decomposition is standard in economic literature since the seminal work of Solow [12], and its mathematical formulation can be derived from the model presented in the Supporting Information. In this setting the GDP per capita growth rate can be written as:

$$\begin{array}{c} y_{c,t}=a_{c,t}+\alpha k_{c,t}+\left(1-\alpha \right)e_{c,t}+\left(1-\alpha \right)h_{c,t}. \end{array}$$ (1)

where *a*_*c*,*t*_ is the growth rate of GDP per capita due to the (exogenous) technological efficiency of the country *c* at time *t*, *αk* the growth due to the increase of physical capital per capita, (1 − *α*)*e* the growth due to an increase of the labor force share in population, (1 − *α*)*h* is the growth due to an increase the human capital (education) of workers of the country, and *α* is the output elasticity of capital, where the output elasticity of labor is assumed to be (1 − *α*). This assumption is related to the return to scale of the economy being constant: doubling both labor and capital will double the output of the economy. While this is obviously not true for firms, where production techniques are very different for small and large firms [13], it is a well established empirical fact when considering countries [14]. These last three addenda form our definition of input growth, being it a physical investment in new machinery (*k*), an increase in labor force participation(*e*), or additional education (*h*).

Since the growth rate of inputs is quantifiable, to compute the different parts of growth in Eq 1 we only need to estimate *α*. Economic theory is handy in this case. If each factor of production is paid for at its marginal value, the share of national income going to capital will be *α* and the share going to labor 1 − *α*. Since these shares are observable numbers, we will use them to estimate *α*. Finally, the efficiency part *a* can be recovered as the residual after removing the inputs component from the total GDP per capita growth.

In the following empirical exercises to understand industrialization we will focus on the growth of GDP per capita due to input growth. Why are we interested in *input* growth, and not in *total* per capita GDP growth? While the overall measure of GDP growth can be deceiving and can be influenced by the price and discovery of natural resources and the occurrence of any external factor, the sudden investments in physical and human capital and the increase in labor force participation are the clear fingerprints of a structural change, a movement from an equilibrium to another.

Much of the academic world focused only on efficiency and productivity, assessing that most of the long term growth is due to productivity growth ([3, 15]). This is obvious: input growth is intrinsically limited. While a country can double its employment rate from 30% to 60% in the first 20 years of industrialization, it cannot double it again in the following 20 years. While a country can quickly increase literacy rate to 90%, further efforts cannot give similar payoffs. Even if physical capital could, in principle, grow without bounds, its effectiveness in terms of increasing labor productivity would decline once technological progress is included in the description. As a consequence, input growth suffers from decreasing returns, and it cannot be the focus for long-term growth in developed countries. However, the growth due to inputs is indeed a powerful force at the onset of a country’s industrialization.

In analyzing, for example, the case of Singapore’s industrialization during its high growth phase, the excess growth required to catch up with the developed world was obtained through input growth. In 1970, the year of Singapore’s maximum growth, out of an impressive 11% growth rate of real per capita GDP, 8% was due to input growth.

Even without decomposing the growth, the transforming effect of industrialization on Singapore is visible by simply looking at the changes in the descriptive statistics in the time span of one generation. In 1966, at the beginning of Singapore’s industrialization, 27% of the country population was employed, and 39% of the new entrants in the job market had no formal education. Only 16% of the new entrants in the job market had at least a secondary degree. Furthermore, investments in physical capital were modest, with only 10% of saving rates. The generation entering the job market in 1990 were confronted with a deeply changed country. Female workers had entered massively the job market, and 51% of the population was now working, almost twice as much as in the previous generation. Among entrants in the job market 10% alone do not have any formal education (one fourth compared with the previous generation) and 54% have at least a secondary degree (a threefold increase). Saving and investing has become common among the population, the savings rate has increased fourfold reaching 39% ([10, 16]). Not surprisingly, in the same time span Singapore’s GDP has increased by almost 9 times and the per capita GDP has increased by almost 6 times. Of this exceptional growth, only a modest 25% (around 1% per year, in line with developed countries), can be assigned to a growth in productivity (data from Penn Table 8.0 [11]). In conclusion, during industrialization the input growth defines the trend of per capita GDP growth, while the residual productivity increase acts mostly as noise over the growth signal.

### An Economic Complexity approach to Growth

As mentioned in the introduction, we describe the industrialization of a country as a non-linear, dynamic, off-equilibrium process in which a complex network interconnecting production capabilities and product demand emerges at the country scale. It has been suggested by [17], that the properties of this complex network of not measurable intangibles, can in fact be characterized by looking at the properties of the international trade network. Indded modern goods markets constitute a network of products similar to that formed by the nodes of the world wide web. For this reason ranking algorithms can efficiently be used to characterize the trade-network properties, and in particular to rank nations according to their manufacturing capabilities and product complexity. The manufacturing capabilities of each country reflects the cultural and technological structure of the economy of that country ([18, 19]), which in turn is related to the basket of products that that particular country is able to produce and export ([20]).

This idea of using PageRank ([21]) type of algorithms to rank countries economies has been pioneered by [17], kindling a rich literature on the subject. In particular [7] applied this view to introduce a novel non-monetary indicator based on the properties of the network formed by interstate goods exchanges in which non-linear interactions play a fundamental role. Since we are interested in the non-linear properties of the emerging network, which alone can accomodate our view of the explosive combinatorial dynamics of the country’s capabilities and demand underlying the industrialization process, in this work we will follow the latter approach.

According to [7] the information of the country economic potential can be extracted from the properties of the worldwide export network, that we take from UN-COMTRADE [22], as expressed by the structure of the matrix **M** whose entries *M*_*cp*_ take the value 1 if the country *c* shows a Revealed Comparative Advantage ([23]) in the export of a (physical) product *p* and 0 otherwise. This choice has a number of advantages: i) in this way any trivial correlation with export volumes is removed allowing comparison of countries of different size; ii) a comparison of countries with very different shares of manufacturing sectors of total GDP is also permitted, since only the country export basket composition is relevant to rank that country economy; and finally, iii) while the matrix “digitalization” in binary form necessarily implies a loss of information, it has been shown that this kind of treatment is far more resilient with respect of the possible presence of noise in the data ([24]).

The products exported by a country are for certain not all equal in terms of the capabilities required to produce them: some products in fact require a higher number of (or more exclusive) capabilities to be manufactured. For this reason it would be too simplistic to assume the country export basket diversification as a measure of the country’s economic efficiency. It is more reasonable to relate this efficiency the complexity of the products produced by that country, defining in turn a product to be complex if it requires a higher number (or a more sophisticated set) of capabilities to be produced.

Indeed what is required is a way to measure, although indirectly, the countries’ capabilities signaled by the production (and export) of a given product. As the capabilities are not directly measurable, we will summarize the capabilities needed to export a single product as a single value, that we call product complexity, *computed only relying on the structure of the country-product network*. Let us suppose for the moment this numeric value to be known. Given these product complexities, the fitness of a country will simply be defined as the sum of the complexities of its exported products (mathematical expression below) and it will be representative of the country’s capabilities, i.e. a measure of the complexity of the country economy. We refer to this quantity as the country fitness. We will show in the following how this measure of fitness empirically relates to the industrialization of the country. In turn, the complexity of a product can be defined through the fitness of the countries exporting them: *a product is considered complex if low fitness countries do not export it*. Consider this simple case: only highly developed countries export transistors, while both more industrialized and less industrialized countries export nails. Therefore the low complexity of nails can be deduced directly by the fact that low fitness countries are also able to produce and export them. As a consequence low fitness countries are more informative in assessing the complexity of products. All these considerations yield *to a crucial non-linear relation between fitness of countries and complexity of products*, which is implemented in the algorithm we use, at difference with that originally proposed by [17].

More formally, to calculate the fitness of countries *F*_*c*_ and the complexity of the exported products *Q*_*p*_ we iterate upon convergence the following set of non-linear coupled equations:

$$\begin{array}{c} {\tilde{F}}_{c}^{\left(n\right)}=\sum_{p}M_{cp}Q_{p}^{\left(n-1\right)} \end{array}$$ (2)

$$\begin{array}{c} {\tilde{Q}}_{p}^{\left(n\right)}=\frac{1}{\sum_{c}M_{cp}\frac{1}{F_{c}^{\left(n-1\right)}}} \end{array}$$ (3)

$$\begin{array}{c} F_{c}^{\left(n\right)}=\frac{{\tilde{F}}_{c}^{\left(n\right)}}{<{\tilde{F}}_{c}^{\left(n\right)}{>}_{c}} \end{array}$$ (4)

$$\begin{array}{c} Q_{p}^{\left(n\right)}=\frac{{\tilde{Q}}_{p}^{\left(n\right)}}{<{\tilde{Q}}_{p}^{\left(n\right)}{>}_{p}} \end{array}$$ (5)

where the normalization of the intermediate tilted variables is made as a second step, n is the iteration index, and the <>_*x*_ symbol stands for the arithmetic mean with respect to the possible values of *x*.

The fixed point of these maps has been studied with extensive numerical simulations and it is found to be stable and not depending on the initial conditions. We refer to [25] for a detailed description of the algorithm and a comparison with the one proposed by [17]. The convergence properties of the fitness and complexity algorithm are not trivial and have been studied in [26]. This methodology has been applied to both the study of specific geographical areas, such as the Netherlands ([27]) and the Sub-Saharan countries ([28]), and general features of growth and development ([9, 29]).

**Fig 1:**
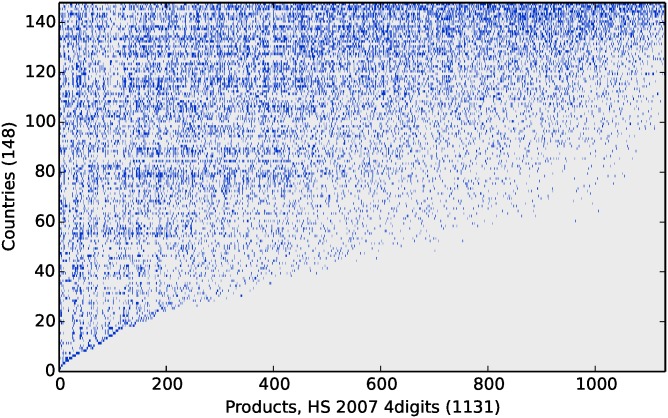
Binary matrix identifying countries producing a specific product. Countries (rows) are ordered according to their Fitness, Products (columns) are ordered according to their Complexity. A clear triangular structure emerges.

Once countries and products are arranged according to their respective fitness and complexity, the matrix **M** is roughly triangular, as shown in Fig 1. This structure implies that developed countries tend to have a highly diversified export basket, since they export both complex and not complex products, while poor or less developed countries export fewer and less complex products. While diversification may lead to an immediate (zero-order) estimate of the fitness of a country commensurate with the number of different products it exports, the evaluation of the products’ complexity is more subtle: a product exported by low-fitness countries should be assigned a lower score since it is reasonable to expect that a lower level of capabilities is required to produce it. Clearly a linear page-ranking type of analysis cannot handle this point, while a nonlinear ranking approach—as the one presented above—will be more appropriate.

## Results

### Empirical findings and their relation to poverty trap

As mentioned in the introduction, while economic theory is mostly related to equilibrium processes, studying balanced growth at the equilibrium ratio, industrialization is obviously a dynamic process occurring between different growth paths, while the country moves from one equilibrium ratio to another. Although growth in equilibrium is driven by the growth in productivity, the idea that there can be multiple equilibrium ratio of balanced growth and a barrier to overcome to shift from one equilibrium ratio to another was already present in [1]. In the transition the country experiences high GDP growth through input growth. There can be a capital barrier, like in [1], or a demand barrier, like in [6]. Still, there is a threshold to overcome in order to access the input driven out-of-equilibrium growth spike. Even in models considering the evolution of a country as a unified process, like [30], there are variables that must reach a tipping point in order to move the society into a high growth regime, driven by incentives to invest in production inputs. Given this, two stylized facts should then be expected to emerge by looking at empirical data:

First, we should expect a certain level of GDP (or physical capital, in Solow’s perspective) per capita to be required to trigger the transition if the barrier to start the industrialization is demand driven as in [5] (or capital driven, in Solow’s perspective [1]). We should therefore find a positive relation between per capita GDP growth due to inputs and the level of per capita GDP for low levels of per capita GDP, where additional per capita GDP means additional internal demand and implies higher per capita physical capital. Second, if the catching up of developing countries is the result of the dynamics of inputs to a new equilibrium, we should expect high input growth among the developing countries, sharply declining for the developed ones. We should therefore expect a negative relation between the GDP growth due to input and the level of GDP for the countries that have started the transition: the growth should slow down for developing countries while the level of inputs approaches the new equilibrium and the developing countries catch up with the developed ones.

**Fig 2:**
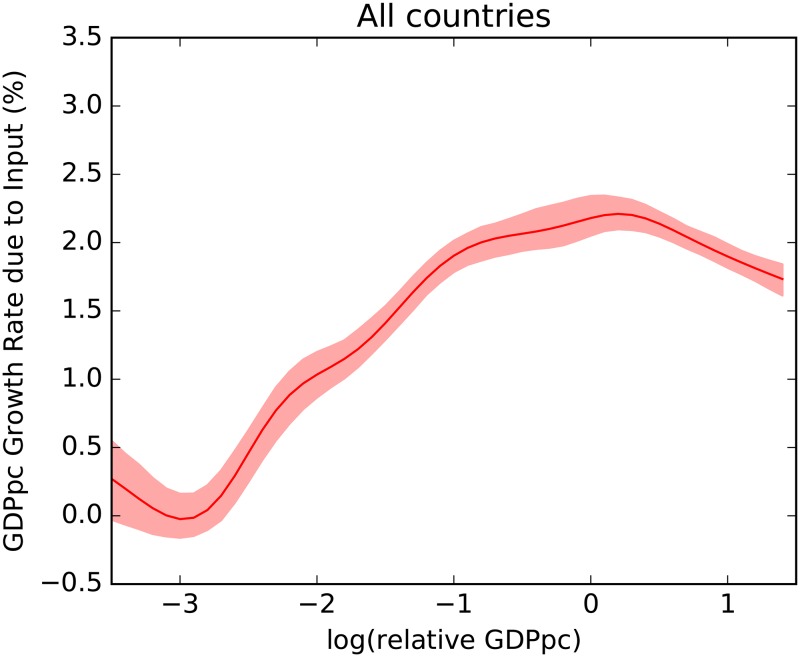
Non parametric kernel estimation of growth rate of per capita GDP due to inputs versus relative per capita GDP. The shadowing indicates 90% of confidence interval of the expected value, computed with bootstrap. Different countries-years in the range 1963–2000 have been pooled after removing the global trend. While the low performance of low GDP countries in increasing their input is clearly visible (left site of the figure), the slowing down of input growth expected after catching-up (right side) is modest.

To check these expected empirical behaviors we compute the average growth rate due to input versus the country related per capita GDP. We do so by pooling all the countries and years. In particular, we will use a non parametric Gaussian kernel estimation [31, 32] to compute the expected value of the per capita GDP growth rate due to input and the corresponding confidence interval for different values of per capita GDP. We plot the results in Fig 2.

The results in Fig 2 do support the existence of a barrier to growth, since there is for sure a certain role played by prosperity to kick-start investments. Data do not seem however to support the second hypothesis: if any catching up mechanism is visible from the data, it is not an awe-inspiring event. The slow down for very high level of GDP per capita, while statistically significant, has poor economic meaning due to the presence of the plateau at medium large levels. For sure it does not support the image of calm after the storm that is expected in a poverty trap model.

Clearly, at this level our analysis is missing a crucial ingredient: we are not able to pinpoint the possibly different growth potentials among countries.

As it is well known, some countries have started an impressive growth process, from an industrial and a social point of view, while others simply rely on the exploitation of natural resources; the empirical disentanglement of these two situations, that are the result of the strategic political choices of countries, will be the subject of this paper. For the same level of physical capital or GDP, two different countries could live a moment of intense investment and shared opportunities for the whole of the population, favoring investments both in physical and human capital, or a moment of stillness and complacence, often characterized by exploitive economic institutions and high inequality. We need a quantitative measure in order to discriminate among these and others situations; from a practical point of view, a new dimension to disentangle different economies, possibly independent from the ones which are usually taken into account in mainstream economics. We believe that concepts taken from the economic complexity approach may be of help. In particular we believe that the fitness of a country, being both a quantitative measure of the number and of the quality of capabilities of a nation and a measure of diversification in advanced and complex products, is a useful indicator of the potential for growth of the country.

**Fig 3:**
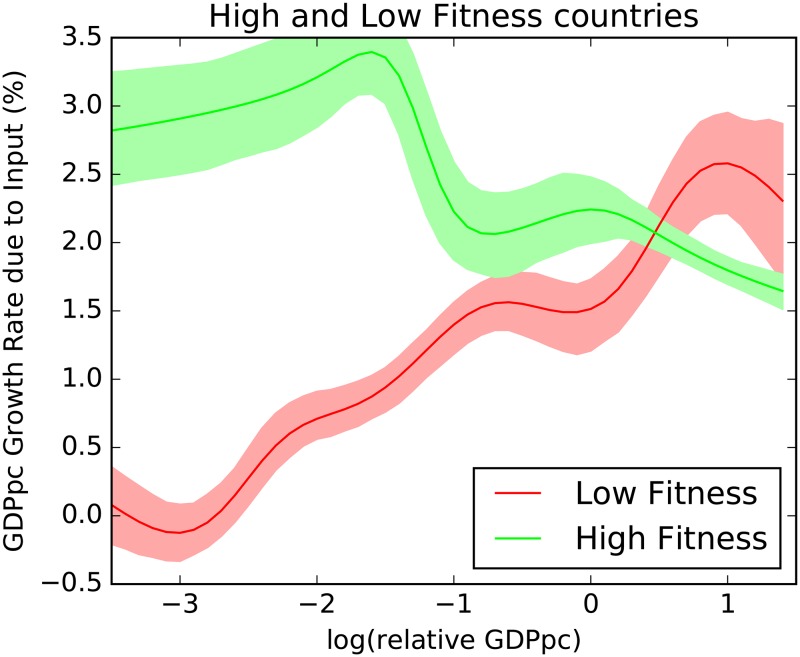
Non parametric Gaussian kernel estimation of growth rate of per capita GDP due to input versus per capita GDP for the lowest and the top tertile of the fitness distribution. The shadowing indicate the 90% confidence interval of the expected value, computed with bootstrap. Different countries-years in the range 1963–2000 have been pooled after removing the global trend. Dividing the countries in three sets of the same numerosity depending on their fitness values highlights very different behaviors and reconciles the theory with the empirical observation.

We will therefore replicate the exercise dividing the countries in three sets of the same numerosity accordingly to their fitness ranking, to see if it helps disentangling the different regimes. In particular, we consider as high fitness countries those which are in the top 33% of the fitness ranking, and low fitness countries the bottom 33%. In Fig 3 we show the results for the high fitness countries compared with the low fitness ones.

When data are split in this way, two different patterns emerge. What was confused when clubbing all the countries together is now visible and in agreement with the theoretical models. The high fitness economies, the ones able to differentiate their production in advanced products, present a clear downward slope of the growth of GDP per capita due to input growth with respect to the level of GDP per capita. The countries with lower fitness instead have issues to start the transition. They experience an higher barrier and they need a very high level of GDP per capita to experience the mobilization of resources expected by economic theory.

The complexity based measure of country fitness seems to lower the barrier to start the transition, and we will see it in more detail in the next session.

### The role of complexity in Economic Growth

**Fig 4:**
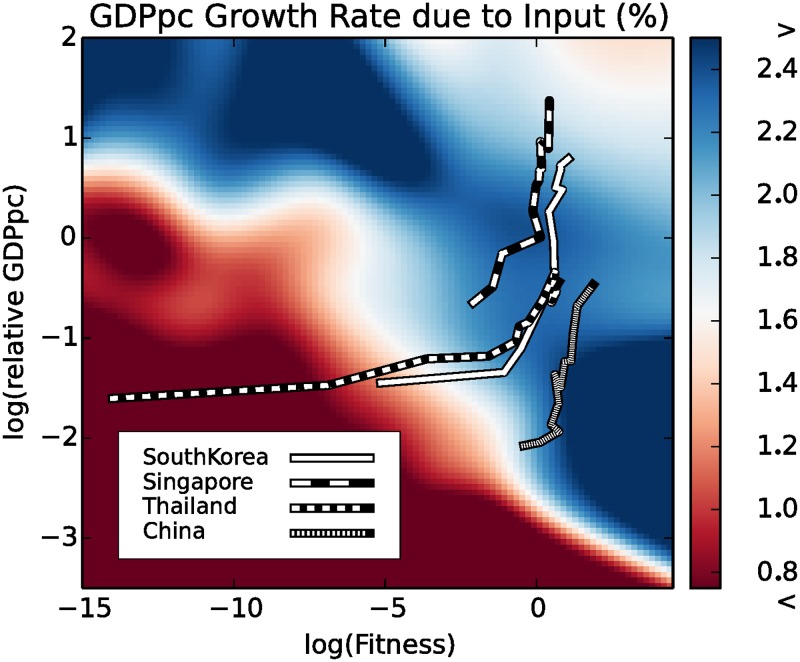
The color map represents the Per Capita GDP Growth due to inputs, for different values of Fitness and GDP per Capita. Different countries-years in the range 1963–2000 have been pooled after removing the global trend. Both the role of the fitness of the country in lowering the threshold to enter in the high endogenous GDP growth regime (the blue band in the center) and the slowing down of the process for developed countries (the top-right corner) are evident.

**Fig 5:**
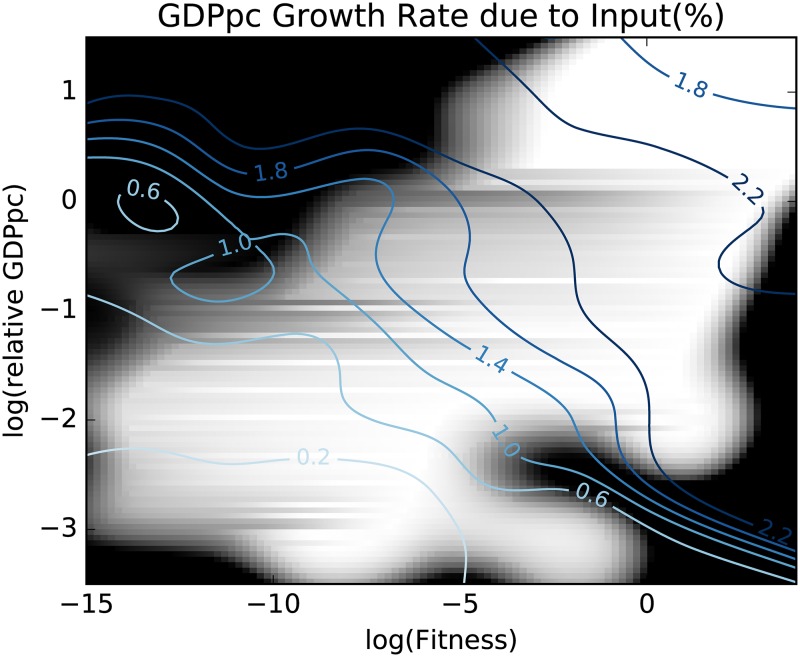
This plot present two bits of information on the expected GDP growth due to input growth in the plane fitness—relative GDP per capita. i) in different shades of blue, the isolevels of GDP growth due to input growth, computed with a spacing of 0.4%; ii) in shades of grays, the estimation error of GDP growth due to input, where black means a standard error of 0.4% or more, and white a standard error of 0.2% or less. Different countries-years in the range 1963–2000 have been pooled after removing the global trend. The figure highlights the complementary role of Fitness and relative GDP per capita to identify the expected value of GDP growth due to input growth.

In the previous section we observed different behaviors for countries with different fitness levels, hinting at a possible explanation for the industrialization process of a country, which is fostered by high fitness levels. The complexity of the country’s economy brings down the barrier to industrialization and allows for investments in inputs. This hypothesis, however, requires further investigation. The different behavior of countries grouped according to their high (gree line) or low (red line) fitness levels (Fig 3), needs to be generalized to a continuous description. This is the aim of the present section in which we compare the growth rate of per capita GDP due to inputs, with both the detrended per capita GDP Y and the fitness *F*. This is achieved again by a non parametric estimation of a two dimensional Gaussian kernel [31, 32] obtained by pooling all the countries and years for the time period in question. At difference with the analysis in Fig 3, in which we compared only the behavior of the highest and the lowest fitness countries, we here explore the complete range of fitness values. This is equivalent to adding a further dimension to the analysis. The results are reported in Fig 4. To represent the three dimensions, the dependent variable, the growth due to inputs, is visualized as a color map. To further explain the relation between the two representations, we notice that the leftmost part of Fig 4 is populated by the same countries belonging to the red line in Fig 3 (low growth potential countries), while the rightmost part is populated by the countries belonging to the green line (high growth potential countries). Fig 4 does not convey any information on the estimation error in different points of the plane: this is particularly tricky since the estimation error is, of course, very heteregenous in different parts of the plane: since fitness and relative GDP per capita are correlated, most of the points are on the diagonal: there are few countries with high fitness and low relative GDP per capita or viceversa, and estimations in those parts of the plane have therefore a wider confidence interval. Therefore, we show in Fig 5 this information by shading the parts of the plane where the standard deviation is larger than 0.4%. The figure also shows the different isolevels of the per capita GDP growth due to inputs in a complementary way to the color map presented in Fig 4.

This result strongly supports our argument: the complexity and diversification of a country’s economy acts as a catalyst in triggering the transition by significantly reducing the necessary per capita GDP. The catching up phenomenon is barely observable in Fig 2 since the plot represents the average of different fitness levels for each level of per capita GDP, thereby mixing up different states of the transition. Even when starting from very low levels of per capita GDP, high fitness countries are able to start the transition, with increasing investments causing increasing input growth levels. On the contrary, low fitness countries characterized by exports concentrated in few low complexity sectors, require very high levels of per capita GDP to start the transition and attract investments. It is trivial to adapt this result to a demand-side explanation: there is a complementarity in kindling the industrialization process of a country between fitness, which is a proxy for export competitiveness, and per capita GDP, which is a proxy for internal demand.

However, even a supply-side explanation is consistent with our empirical results, since the opening of new export sectors increases the incentives to invest. One would expect this increase in incentives to be even more visible looking at the residual productivity growth, *a*_*ct*_. However, if the new accessible sectors allow new—intrinsically different—inputs to be used and accumulated, the scale of production can increase without a corresponding increase in the factor productivity, consistently with an input driven growth. Low fitness countries with poorly diversified economies do not start the endogenous transition until they have reached an extremely high level of capital, staying stuck in the so-called poverty trap. In this sense the poverty trap can be seen as a 2 dimensional object in which both the population wellness (per capita GDP) and the complexity of the national economy (country fitness) play a role.

A different point of view to explain the same empirical data would be a demand side explanation imagining a continuous transition between internal and external demand, proxied respectively by the GDP per capita and the fitness of the country, that is a measure of the complexity and value added of the exported goods.

Finally we add some comments about the trajectories of the countries we considered in Fig 4. The depicted countries are an the extended set of Asian Tigers, originally including South Korea, Singapore, Hong Kong and Taiwan, a set of countries well-known for their impressive pattern of industrialization. In particular, South Korea and Thailand represent a paradigm for what we intend with a *lateral escape* from poverty trap. Taking advantage of the two-dimensional structure of the trap, they have increased their fitness first, and when their economy reached a high level of complexity they started the upward movement towards a balanced and richer society, reaching a higher level of GDP per capita. The careful reader will note that the case of China is complementary: even if the fitness of China was high even in the sixties, the extreme poverty of a large share of its population prevented this country to overcome the barrier and start to grow in a sustained way. From this point of view, the industrialization of China from the sixties to nowadays can be described in terms of the neoclassical poverty trap, in which only one dimension is needed.

In this perspective it comes natural to try to define a new base in the Fitness—relative GDP per capita plane, such that the process of industrialization and the escape from the poverty trap can be described of a single dimension. This will be the target of the next section.

### Defining a Complex Index of Relative Development

**Fig 6:**
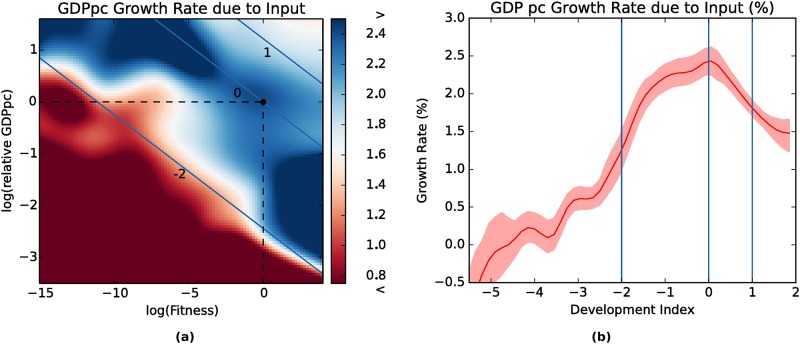
In Fig (a), color map of the per capita GDP growth due to input in the Fitness—relative GDP per capita space, as in Fig 4. Superimposed to it, isolevel lines of *CIRD_c,t_*. In the figure we also highlight the origin, corresponding to an hypothetical country having average GDP per capita and Fitness. In Fig (b), non parametric kernel estimation of per capita GDP growth due to input versus *CIRD_c,t_*. The shadowing represent 95% confidence intervals. The blue lines correspond to the same lines of Fig (a).

Fig 4 and—particularly—Fig 5 also highlights the linear form of the complementary role between the logarithm of Fitness and the logarithm of relative GDP per capita in explaining development. This leads to the natural definition of an index of development and industrialization that we call “Complex Index of Relative Development” as geometric combination of fitness and GDP per capita:

$$\begin{array}{c} CIRD_{c,t}=\log\left(F_{c,t}^{\beta }GDPpc_{c,t}^{1-\beta }\right)=\beta \log F_{c,t}+\left(1-\beta \right)\log GDPpc_{c,t}. \end{array}$$ (6)

where suffix *c*,*t* indicate country *c* at time *t*. Of course the assumption that the coefficient *β* and 1 − *β* of Fitness and relative GDP per capita sum to 1 does not have any economic implication, since the *CIRD* is not a directly observable quantity and therefore any monotonous transformation would be unconsequential. In the following, *β* equal to 0.18 has been used simply to renormalize accordingly to the different span of the logarithms of relative GDP per capita and Fitness which are of course dependent on the specific time span and database. The index is represented in Fig 6. In the figure we show how the use of the index allows to study once again development as a monodimensional process.

The idea that development is related to a combination of GDP per capita and a measure of capabilities immediately summons into mind the Human Development Index developed by United Nations Development Program (UNDP) [33] embodying Amartya Sen’s “capabilities” approach [34]. However in this case the capabilities are measured in a data driven algorithmic way, without the arbitrary definition of a combination of education and life expectancy. In this light the capabilities are not simply an objective, a measure of well being, but also a mean for further development. We see how there is a sharp prediction of a threshold to exit the poverty trap at *CIRD*_*c*,*t*_ ≈ −2, −2 being the flex point of the curve in Fig 6(b) (that is, the point in which the increase of the input growth reaches its maximum). As enhanced by the threshold between the red and the blue area in Fig 6(a), it is also a value of the input growth which is similar to the one of the developed countries. Notice that, given the definition in Eq 6, a *CIRD*_*c*,*t*_ = −2 means that the country *c* at time *t* differs from the world average both in GDP per capita and Fitness of a factor e^2^ ≈ 7.4, or a proportionate combination of the two.

The values of the index for the countries and the years used in the analysis is presented as supplementary material, as S1 File. Values for further years are available from the authors upon request.

Finally, we would like to stress that these results are robust with respect to reasonable changes in the databases ant the time span. In particular, in the Supporting Information S1 Appendix we present similar results, obtained on a much more recent database which refers to about 20 and not to 50 years like the one used in this section.

## Discussion

We showed in the paper that simple toy models of countries’ growth (in particular models assuming that all countries are homogeneous objects characterized only by one state variable, being it the per capita GDP or per capita physical capital) are not able to capture the different patterns of industrialization and, therefore, to predict the starting point of industrialization, that is the moment in which the countries will escape from the poverty trap. We also showed that the measure of Fitness is able to properly disentangle these different patterns, highlighting countries that are ready to take off and to industrialize and countries that, even with similar standards of living, are far from the threshold.

These findings suggest a possible role for the complexity of the economy as a driver for opportunities and for attracting internal or external sources of investment. The increased number of complex production sectors, proxied by the fitness, leads to an open array of possibilities allowing individuals to invest in physical and human capital in order to exploit new and additional opportunities. This effect of fitness on savings and education could be modeled in terms of a dynamical process in which a multi-sector economy goes from one equilibrium to another after overcoming a threshold that can be lowered by increasing the complexity and diversification of the economy. We believe that this out of equilibrium dynamics is the one performed by countries emerging from the poverty trap. Further details on the possibility to formally describe such dynamics can be found in the Supporting Information.

This analysis does not scrap the concept of the poverty trap: if anything, it makes the original point stronger. As can be seen in Figs 4 and 6(a), not only the barrier is real but, in countries with a low fitness, it is extremely high. To the extent of the apparent paradox that a low fitness country in order to emerge from the poverty trap must first become rich. However, it also suggests a different way to overcome both the trap and the paradox, lowering the threshold to industrialization by diversifying production and exports and making its economy more complex.

An analogy can be drawn between this subtle, country-dependent interplay between fitness and GDP per capita and phase transitions, such as the transition of water from the liquid state to the gaseous one. When water is heated, in general, its temperature increases. This is analogous to a country which is already out of the poverty trap, and whose efforts are entirely reflected in an increase of its GDP per capita. However, during a phase transition the absorbed heat is used to break the bonds among the molecules, and even if the temperature does not increase, this process is fundamental to change phase. Similarly, when a country exits from the poverty trap by increasing its fitness the consequences of its efforts do not give immediate results in terms of GDP per capita, but they are firmly grounded in the requirements for a future, sustained growth.

Our analysis is particularly relevant for policy makers interested in the first steps of development, in particular for the ones interested in countries that are unable to complete their industrialization process even if they enjoy moderately good standards of living thanks to the presence of natural resources. If the pursuit of an industrialization process through further input accumulation is not feasible, developing the country capabilities to allow for more diversified production, particularly in complex products, can be the key to escape the poverty trap.

The introduction of the new Complex Index of Relative Development (CIRD) helped us to understand the industrialization dynamics and may be as helpful for policy makers and other acadmic and institutional stake holders to implement such insights in their development analysis.

Finally, we mention some future directions of research. One would like to understand how the possible overcoming of the industrialization threshold introduced in the present paper depends on various countries’ features or endogeneous events, such as income inequality [35, 36] and investments in basic research [37].

## Supporting Information

S1 Appendix Dataset, Basic Solow Model of Growth and Growth Accounting, Robustness checks with the 1995–2010 database. This appendix has three roles: i) to describe the characteristics of the datasets used; ii) to explain a basic Solow Model, necessary to understand how to empirically account the growth components, dividing the total GDP growth in growth due to input growth and exogeneous growth; iii) to do a further robustness check, testing the main results of the paper for a different dataset and a different time period.
(PDF)

S1 File CIRD Values. The values of the CIRD index for all the nations and years used in the paper.
(CSV)
