# Supplementary material for: Complex Economies Have a Lateral Escape from the Poverty Trap
Source: PLoS One. 2017 Jan 10;12(1):e0168540. doi: 10.1371/journal.pone.0168540 (PMC5224870; doi:10.1371/journal.pone.0168540)
Supplement: S1 Appendix — This appendix has three roles: i) to describe the characteristics of the datasets used; ii) to explain a basic Solow Model, necessary to understand how to empirically account the growth components, dividing the total GDP growth in growth due to input growth and exogeneous growth; iii) to do a further robustness check, testing the main results of the paper for a different dataset and a different time period. (PDF) [file pone.0168540.s001.pdf]

Complex economies have a lateral escape from the poverty trap: Appendix

Emanuele Pugliese*[1,2], Guido L. Chiarotti[1,2], Andrea Zaccaria[1,2], Luciano Pietronero[2,1]

1 Institute for Complex Systems - CNR, Via dei Taurini 19, 00185, Rome, Italy
2 Dipartimento di Fisica, Sapienza Università di Roma, P.le Aldo Moro 2, 00185, Rome, Italy

*** emanuele.pugliese@gmail.com**

Dataset

In this work we use mostly simple national statistics, other than the Fitness measure described in the main text.

We use for all the national statistics data the Penn World Table 8.0. [4] The data on physical capital is produced with a perpetual inventory method, as described in [6], while the proxy for the human capital is the average number of years of education in the population. The exogenous growth has been computed as the residual of the total growth after removing the input growth.

Countries' fitness evaluations are based on the import-export flows as registered in the UN-NBER database, reconstructed and edited by [1]. This database includes the (discounted) imports of 72 countries and covers more than 2577 product categories for a period ranging from 1963 to 2000. Exports are reconstructed starting from these imports, which cover about the 98% of the total trade flow. There are many possible categorizations for products; we will base our study on the Sitc v2, 4-digits coding. After a data cleaning procedure, whose aim is to remove obvious errors in the database records, and to obtain a consistent collection of data, the number of countries fluctuates between 135 and 151 over the years, while the number of products remains equal to 538.

The years and nations used in the analysis are reported in the supplementary materials along with the Complex Index of Relative Development value for each year.

Basic Solow Model of Growth and Growth Accounting

In this appendix we give a simple version of the models of economic growth based on [2] and [5]. Even if the aim of the paper is an empirical analysis, we thought it might be useful to the potential reader new to the macroeconomic analysis to have a simple version of economic model aimed at explaining growth. Moreover this derivation is required in our analysis to decompose the growth in input growth and exogenous growth.

We start writing a production function as generic as possible,

$$Y_{c,t} = Y(A_{c,t}, I^j_{c,t}), \tag{1}$$

where $Y_{c,t}$ is the production of country c at time t, $A_{c,t}$ is an efficiency measure and, for different js, $I^j_{c,t}$ are the different inputs of the production (Physical Capital, Labor, Human Capital, ...). The production function gives the output of the economy for different levels of inputs and efficiency. The growth of output, that we will identify with GDP in the following,

can therefore be the consequence of an efficiency and technological growth, i.e. a growth of A, or an input growth.

In growth models some inputs are accumulated in an endogenous way: a part of the output is invested to build new physical capital, a part of the working time of the laborers is spent to train new workers and accumulate human capital. Their level in equilibrium is the result of their accumulation and their depreciation. Growth due to input accumulation is therefore called endogenous growth. On the opposite side, growth due to technology and efficiency, a growth of A, is called exogenous growth.

In the following we will use a minimal case. We will take the standard Cobb-Douglas production function with the combination of two inputs, physical capital $K_{c,t}$ and labor $L_{c,t}$:

$$Y_{c,t} = A_{c,t} K_{c,t}^{\alpha} L_{c,t}^{1-\alpha}. \tag{2}$$

If a fraction s of the output Y is invested in the production of new physical capital K and a fraction δ of K decades at each time step due to depreciation, the time evolution of physical capital is

$$K_{c,t+1} = sY_{c,t} + (1-\delta)K_{c,t}. \tag{3}$$

Since in this simple model $A_{c,t}$ and $L_{c,t}$ are assumed to be exogeneous processes (for a case in which the technological progress is seen as an endogenous process see [3, 7]), we will have as the only endogeneous variable $K_{c,t}$. Notice therefore that any equilibrium we find in these coupled equations for $K_{c,t}$ is actually an equilibrium path, a value $K^*(A_{c,t}, L_{c,t})$ such that $K_{c,t} = K^*$ do not change if the exogeneous variables do not change. Therefore, since the equilibrium point K^* depends on $A_{c,t}$ and $L_{c,t}$, even at the equilibrium $K_{c,t}$ will still grow if those exogeneous variables grow.

In Fig 1(a) we show that this equation has only one stable equilibrium path. The equilibrium is possible due to the decreasing returns on capital: the more capital a country has, the less output the country gains with an additional unit of capital.

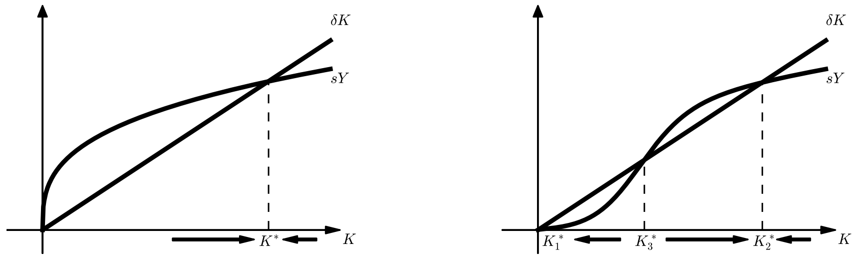

(a) (b)

Figure 1. (a): a single equilibrium in K^*. The two curves δK and sY represents respectively capital depreciation and capital accumulation. The equilibrium is achieved when the two effects are equal, i.e. K^*. (b): a case with multiple equilibria. When the production function has increasing returns for some scales, multiple equilibria are possible. K_1^* and K_2^* are stable equilibria, while K_3^* is unstable.

However already in [2] there were the idea that multiple equilibria in the capital levels are possible. If for example the investing rate s is not independent from the per capita income of the country, but it depends on the achievement on a minimal level of subsistence, the capital accumulation function becomes non linear. We can for example assume a functional form of the investing rate like,

$$s_{c,t} = \frac{s_c}{1 + e^{K_F - K_{c,t}}} \tag{4}$$

where s_c is a country-dependent parameter and K_F a minimum threshold to achieve subsistence and start investing. As a consequence of this non linearity the system can present a behavior similar to Fig 1(b).

After overcoming a barrier, in Fig 1(b) represented by point K_3^*, the country capital would move endogenously to K_2^*. The out-of-equilibrium dynamics from one equilibrium to another has to be characterized by fast input accumulation and, therefore, fast endogenous economic growth. A way of looking the results of our empirical analysis (see the Results section) is that K_F depends on the Fitness of the country.

In this setting we can also understand the decomposition

$$y_{c,t} = a_{c,t} + \alpha k_{c,t} + (1-\alpha)e_{c,t} + (1-\alpha)h_{c,t}. \tag{5}$$

From 2, the GDP per capita is equal to

$$\left(\frac{Y_{c,t}}{P_{c,t}}\right)_{c,t} = A_{c,t}\left(\frac{K_{c,t}}{P_{c,t}}\right)^{\alpha}\left(\frac{E_{c,t}}{P_{c,t}}H_{c,t}\right)^{1-\alpha}, \tag{6}$$

where L is now written as E, the number of employees, times H, a factor related to the average human capital of the work force, and both sides of the equation are divided by the total population P of the country. Therefore, defining with the lowercase letters the growth rates of the respective uppercase variables and with the hat the division by population,

$$\hat{y}_{c,t} = a_{c,t} + \alpha\hat{k}_{c,t} + (1-\alpha)\hat{e}_{c,t} + (1-\alpha)h_{c,t}. \tag{7}$$

In equation 5 we dropped the hats notation to simplify the reading, and y, k, and e are presented directly as the GDP per capita, physical capita per capita, and employment rate.

Robustness checks with the 1995-2010 database

A natural question is whether our results are robust when considering other time ranges and, as a consequence, different countries that experience the industrialization process and the catching up. As a consequence, in this appendix we reproduce the main results for a different database, described in [8], which covers a time span which ranges from 1995 to 2010 (the results in the main text being obtained with the database described in [1], which covers the period 1963-2000). Given the shorter time period (which has been approximately reduced to one third with respect the previous analysis), we would expect a noisier structure but, as we will see in the following, this is not the case, establishing that our results are not only sound but also replicable for smaller datasets.

In Fig 2 we replicate the same analysis of Fig 2 of the main text. One can easily see the different performance, in terms of input growth, of high fitness countries with respect to the low fitness countries at a low level of GDP per capita. After the catching up, on the contrary, the input growth becomes very similar.

The next step is to study the dependence of the input growth from all the fitness ranges, that is, to go from a discrete classification in high and low fitness countries to the study of all the fitness spectrum. This analysis leads to Fig 4 of the main text, and to Fig 3 for the 1995-2010 database. Here too one finds that a high fitness value is associated with a lower monetary threshold to enter in the high endogenous GDP growth regime and start the industrialization process.

Finally, we present in Fig 4 the same analysis we performed for Fig 5 of the main text, for the new database. In this plot we shade those areas in the Fitness-Income plane on the basis of the statistical error on the input growth, and we plot the contour lines corresponding to the different isolevel of the same input growth. Once again, the results are consistent with the ones we discussed in the main text: there is a well defined diagonal area, where most countries lie, in which the input grows steadily from the poverty trap, on the bottom-left corner, to the top-right corner, where the developed countries are.

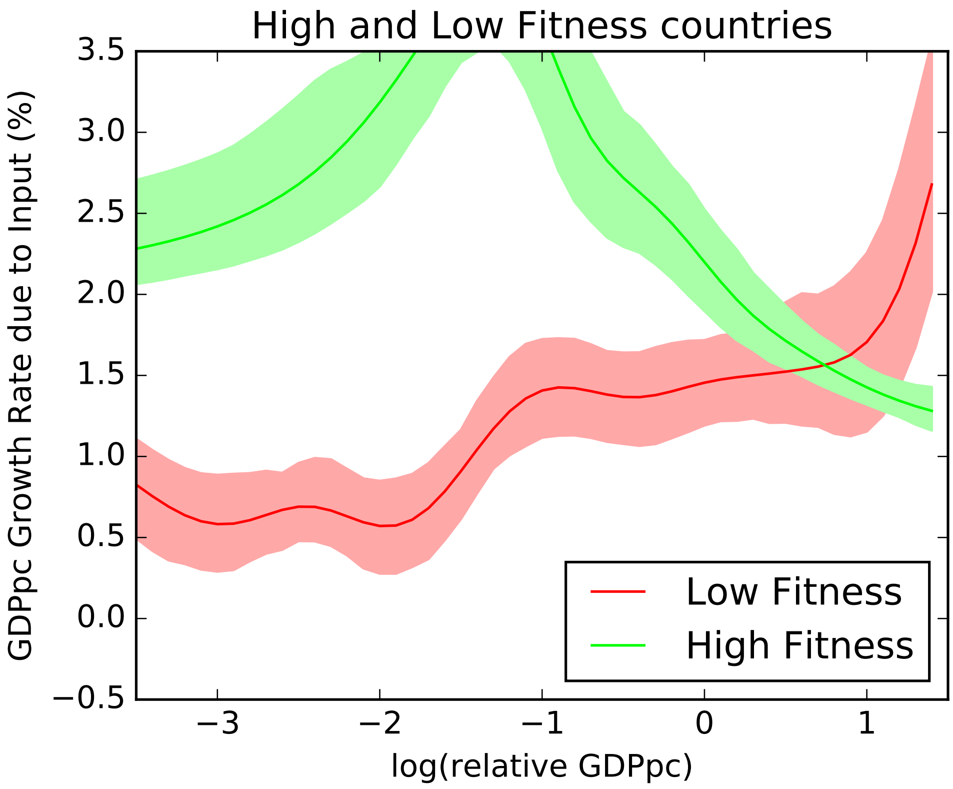

Figure 2. Non parametric kernel estimation of growth rate of per capita GDP due to inputs versus relative per capita GDP. Different countries-years in the range 1995-2010 have been pooled after removing the global trend. The results are consistent with the ones obtained in Fig 2 of the main text.

Acknowledgments

The authors acknowledge funding from the "CNR Progetto di Interesse CRISIS LAB" (http://www.crisislab.it) and EU Project no. 611272 GROWTHCOM (http://www.growthcom.eu) The funders had no role in study design, data collection and analysis, decision to publish, or preparation of the manuscript. We thank Matthieu Cristelli, Andrea Tacchella and Masud Cader for useful discussions and Fabio Saracco for discussions and data processing.

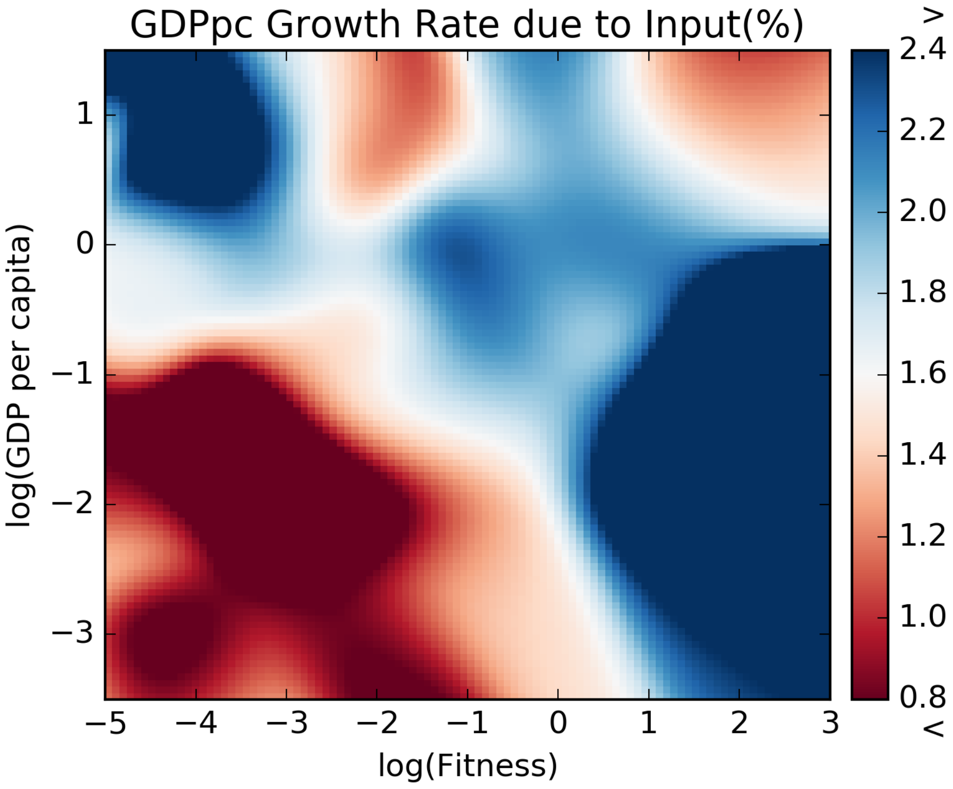

Figure 3. The color map represents the Per Capita GDP Growth due to inputs for different values of Fitness and GDP per Capita. This figure is similar to Fig 4 of the main text, but now the analysis is repeated on the 1995-2010 database. Once again, both the role of the fitness of the country in lowering the threshold to escape from the poverty trap (the white/blue band along the diagonal) and the slowing down after the catching up (the top-right corner) are evident.

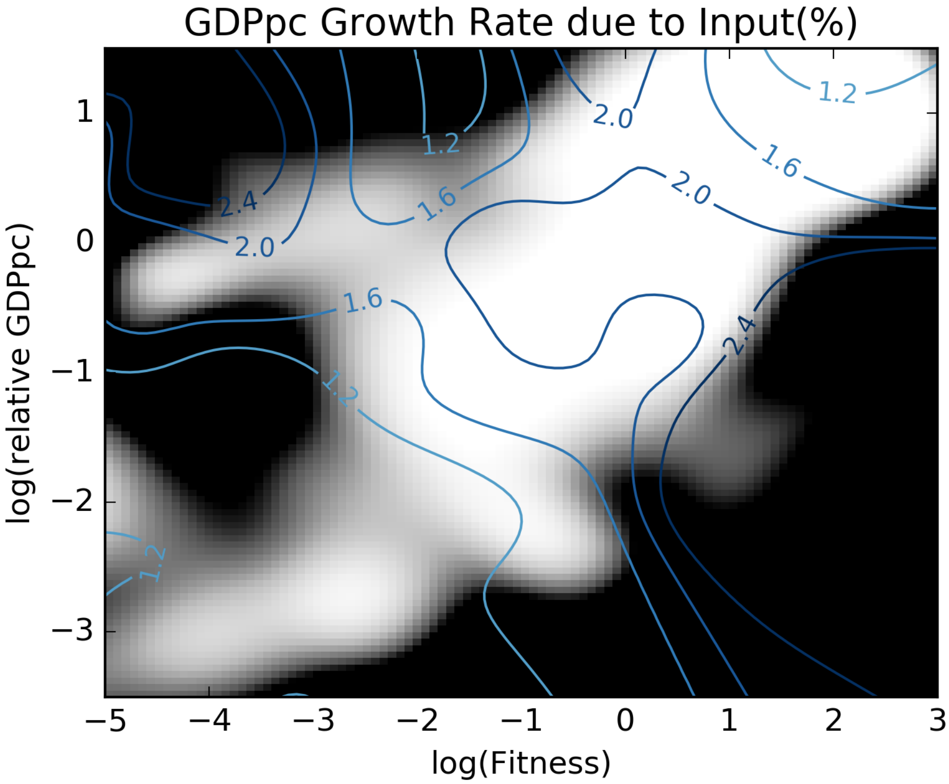

Figure 4. The counterpart of Fig 5 of the main text for the 1995-2010 database. We plot in blue the isolevels of GDP growth due to input growth, ans in shades of grays, the estimation error of GDP growth due to input, where black means a standard error of 0.4% or more, and white a standard error of 0.2% or less.

References

1. Feenstra RC, Lipsey RE, Deng H, Ma AC, Mo H. World Trade Flows: 1962-2000. National Bureau of Economic Research; 2005. 11040. Available from: http://www.nber.org/papers/w11040.

2. Solow RM. A contribution to the theory of economic growth. The quarterly journal of economics. 1956;70(1):65–94.

3. Romer PM. Endogenous Technological Change. Journal of Political Economy. 1990;98(5 pt 2).

4. Feenstra RC, Inklaar R, Timmer MP. The Next Generation of the Penn World Table. The American Economic Review. 2015;105(10):3150–3182.

5. Solow RM. Technical Change and the Aggregate Production Function. The Review of Economics and Statistics. 1957;39(3):312–320. Available from: http://www.jstor.org/stable/1926047.

6. Inklaar R, Timmer M. Capital, Labor and TFP in PWT8. 0. University of Groningen (unpublished). 2013;.

7. Galor O, Weil DN. Population, technology, and growth: From Malthusian stagnation to the demographic transition and beyond. American economic review. 2000;p. 806–828.

8. Gaulier G, Zignago S.

   Baci: International trade database at the product-level. 2010;.
